# The Development and Validation of a Generic Instrument, QoDoS, for Assessing the Quality of Decision Making

**DOI:** 10.3389/fphar.2016.00180

**Published:** 2016-07-13

**Authors:** Ronan Donelan, Stuart Walker, Sam Salek

**Affiliations:** ^1^Global Regulatory ScienceQuintiles, Dublin, Ireland; ^2^Centre for Innovation in Regulatory ScienceLondon, UK; ^3^Department of Pharmacy, Pharmacology and Postgraduate Medicine, University of HertfordshireHatfield, UK; ^4^Institute for Medicines DevelopmentCardiff, UK

**Keywords:** QoDoS, decision-making tool, quality decision, regulatory decisions, submission decisions

## Abstract

**Introduction:** The impact of decision-making during the development and the regulatory review of medicines greatly influences the delivery of new medicinal products. Currently, there is no generic instrument that can be used to assess the quality of decision-making. This study describes the development of the Quality of Decision-Making Orientation Scheme QoDoS^©^ instrument for appraising the quality of decision-making.

**Methods:** Semi-structured interviews about decision-making were carried out with 29 senior decision makers from the pharmaceutical industry (10), regulatory authorities (9) and contract research organizations (10). The interviews offered a qualified understanding of the subjective decision-making approach, influences, behaviors and other factors that impact such processes for individuals and organizations involved in the delivery of new medicines. Thematic analysis of the transcribed interviews was carried out using NVivo8® software. Content validity was carried out using qualitative and quantitative data by an expert panel, which led to the developmental version of the QoDoS. Further psychometric evaluations were performed, including factor analysis, item reduction, reliability testing and construct validation.

**Results:** The thematic analysis of the interviews yielded a 94-item initial version of the QoDoS^©^ with a 5-point Likert scale. The instrument was tested for content validity using a panel of experts for language clarity, completeness, relevance and scaling, resulting in a favorable agreement by panel members with an intra-class correlation coefficient value of 0.89 (95% confidence interval = 0.56, 0.99). A 76-item QoDoS^©^ (version 2) emerged from content validation. Factor analysis produced a 47-item measure with four domains. The 47-item QoDoS^©^ (version 3) showed high internal consistency (*n* = 120, Cronbach's alpha = 0.89), high reproducibility (*n* = 20, intra-class correlation = 0.77) and a mean completion time of 10 min. Reliability testing and construct validation was successfully performed.

**Conclusion:** The QoDoS^©^ is both reliable and valid for use. It has the potential for extensive use in medicines development by both the pharmaceutical industry and regulatory authorities. The QoDoS^©^ can be used to assess the quality of decision-making and to inform decision makers of the factors that influence decision-making.

## Introduction

Decisions needed in the development of new medicines commonly have to be made based on insufficient data, a high degree of uncertainty and significant economical stakes and often occur in an environment of time pressure, in which several stakeholders are competing to be the first in the market with their specific drug candidate (Pritchard et al., 2003; Chung-Stein, 2011). Decision-making among the regulators of medicines and the pharmaceutical industry is driven by various factors. The regulators must adhere to a remit to positively impact public health whilst remaining mindful of precedents and adhering to laws, regulations, and policies (Eichler et al., 2008; Breckenridge et al., 2011). The pharmaceutical industry, on the other hand, is motivated by the need to predictably and transparently develop medicines that will fulfill patients' needs and regulatory requirements whilst delivering profit to shareholders (Breckenridge and Woods, 2005). Additionally, it is recognized that there is a subjective human component within the decision-making process (Donelan et al., 2015). This subjective decision-making style reflects the combination of how an individual perceives and comprehends stimuli and the general manner in which he or she chooses to respond to it. It is linked to an individual's knowledge, ability, motivation, their value orientation and tolerance for ambiguity (Kahenman, 2011).

A review of the literature demonstrates that research and insight into the decision-making approaches and considerations by individuals and organizations involved in medicines research and development is currently lacking. A novel qualitative study investigated the factors influencing quality decision-making (regulatory and pharmaceutical industry perspectives) (Donelan et al., 2015). The outcomes of this qualitative research not only identified the hallmarks of good decision-making practices that could be adopted by all stakeholders at the individual and/or organizational level, but it also emphasized the different focus of the decision-making undertaken by the stakeholders and provided the informed impetus for the development of the generic tool (the QoDoS^©^). An enhanced appreciation may facilitate a clearer understanding of decision-making approaches and this in-turn could help to identify or enable improved decision-making practices for both the individual and the organization. It was this setting that stimulated the research that ultimately led to the development and validation of a generic decision-making instrument, the Quality of Decision Orientation Scheme (QoDoS), to assess the quality of decision making.

## Methods

**Figure 1:**
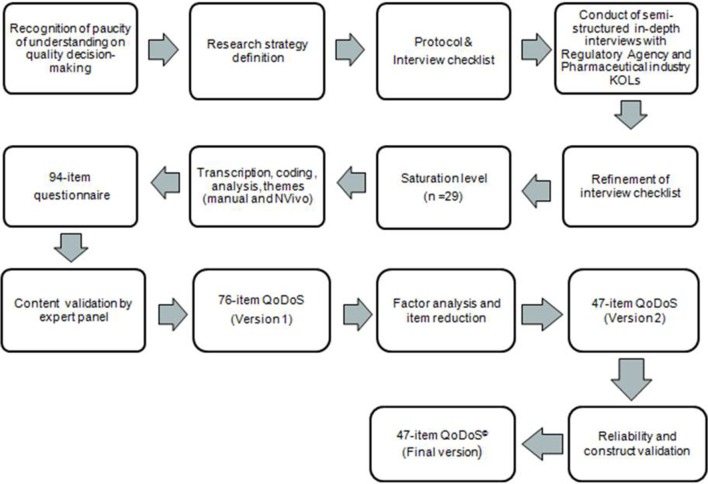
**Development of the QoDoS^©^ decision-making instrument**.

**Figure 2:**
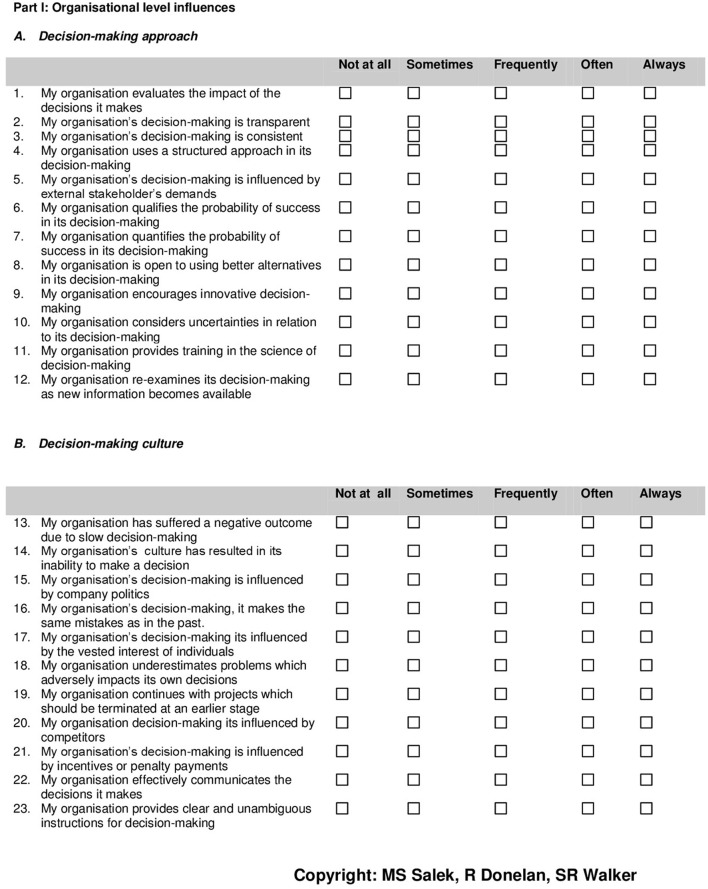
**Quality of Decision-Making Orientation Scheme (QoDoS)^©^. questionnaire: organizational-level influences**.

**Figure 3:**
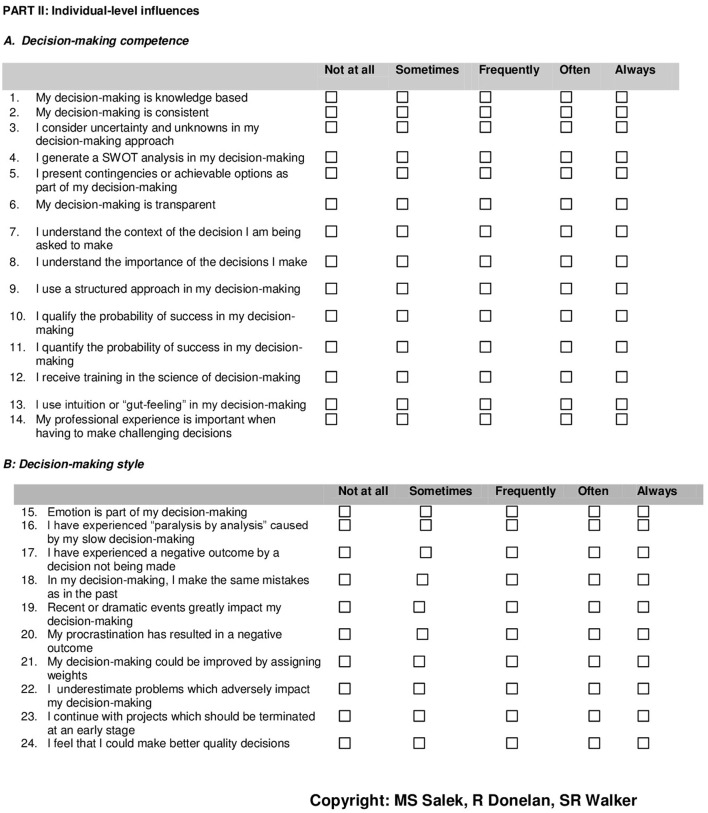
**Quality of Decision-Making Orientation Scheme (QoDoS)^©^. questionnaire: individual-level influences**.

A mixed-methods research approach was adopted comprising both qualitative and quantitative components and considered appropriate for a study of this nature (Hanson et al., 2005). An outline of the development stages of a decision-making instrument is shown in Figure 1. The developmental version of the instrument went through several stages of refinement resulting in a 47-item final version of the QoDoS^©^ (Figures 2, 3). The main stages of the research were; item generation from qualitative interviews, content validation, psychometric evaluation, factor analysis, item reduction, reliability testing and construct validation.

### Item generation

Ninety-four items were generated from the decision-making themes and sub-themes that emerged from the qualitative interviews with 29 senior decision makers regarded as key opinion leaders from the pharmaceutical industry and regulatory authorities to form the first version of the instrument (Donelan et al., 2015). Each item measured one concept in a clear and concise manner. Content validation and psychometric evaluation testing were performed, resulting in a reduced list of items in the form of the 76-item QoDoS^©^ (version 2) questionnaire. This then underwent further item reduction using factor analysis, reliability testing and construct validation, resulting in the final 47-item QoDoS (version 3) instrument.

### Participants: qualitative phase

#### Interviews stage (cohort 1)

Twenty-nine senior decision makers [9 Regulatory Agency; 10 Pharmaceutical Industry; 10 Clinical Research Organization's (CROs)] regarded as key opinion leaders from the European Union (EU) and United States pharmaceutical industry, EU regulatory authorities and contract research organizations (CROs) were interviewed. The sample was chosen because of their active engagement and recognized status in the development, review and delivery of medicinal products.

#### Content validation stage (cohort 2)

A six-member expert panel of experienced senior decision makers from regulatory agencies and the pharmaceutical and CRO industry carried out the content validation on the initial 94-item QoDoS^©^. The pharmaceutical industry (two) and CRO (two) panel members were all experienced professionals at advanced managerial level and all with more than 7 years' experience (range = 7–30 years). The regulatory agency experts (two) were experienced “assessors” with more than 7 years' regulatory agency experience. All content validation panel members considered themselves experienced and experts in decision making.

Each expert member of the content validation panel participated in two ways: first, by individually completing the 94-item developmental questionnaire and the rating of each item using a 4-point Likert scale. Second, by participating in an all panel roundtable discussion meeting once all six feedback forms had been analyzed.

In the content validation, intra-class correlation coefficient (ICC) and reliability (Cronbach's alpha) measurements were determined using SPSS20 statistical software (Pallant, 2005; IBM, 2015). The content validity index (CVI) and scale content validity index (S-CVI) were also calculated for the expert panel review results using Microsoft Excel®. The CVI can be calculated on an item level (I-CVI) and scale level (S-CVI). The item content validity index (I-CVI) is calculated as a level of agreement among a panel of judges for each individual item; that is, the proportion of experts who rate it as content valid. The scale content validity index (S-CVI) is defined as “the proportion of total items judged content valid” (Lynn, 1986; Polit and Beck, 2006). The 76-item QoDoS^©^ (Version 2) was the outcome of the content validation.

### Participants: quantitative phase

#### Factor analysis and item reduction stage (cohort 3)

A sample of 120 individuals from the pharmaceutical industry (*n* = 76), regulatory authorities (*n* = 21), CROs (*n* = 23) from EU, US, Middle East and Singapore participated in the quantitative factor analysis and item reduction phase. This sample was broad, representing the target population.

There were two distinct research component phases involved in the factor analysis and item reduction stage. In component phase 1, a study was conducted using the QoDoS® (version 2) in a large sample to investigate the decision-making approach of individuals and their respective organizations. All participants were asked to complete and return the electronic version of the QoDoS^©^ (version 2). In component phase 2, the quantitative data generated in phase 1 was transposed into statistical format ready for factor analysis and item reduction (Cattell, 1978).

#### Construct validation and reliability testing stage (cohort 4)

The QoDoS^©^ instrument that had undergone factor analysis was evaluated for reliability and construct validity using 76 individuals from regulatory agencies and the pharmaceutical healthcare arena. All participants were asked to respond to each of the QoDoS^©^ 47-items using a 5-point Likert scale: 0 = not at all, 1 = sometime, 2 = frequently, 3 = often 4 = always.

The additional 76 individuals from the pharmaceutical industry (46), regulatory authorities (18) and contract research organizations (12) from Europe, US and China were recruited for this stage. This was a diverse sample representing the target population.

### Statistical analysis

Statistical analyses formed the basis of the construct validation and assessed the correlations between the QoDoS^©^ construct domains. The reliability and construct validity were tested using standard statistical techniques including the Principal Component Analysis (PCA), Cronbach's alpha for reliability accompanied by ICC coefficient and Spearman's 2-tailed correlation statistic (Floyd and Widaman, 1995). Kaiser-Meyer-Olkin (KMO) and Bartlett's Sphericity testing and correlation statistics were also performed. A multi-trait multi-method (MTMM) table was generated capturing comparative statistical methodologies.

### Ethical approval and consent

Because of the nature of the research (interviews and surveys with senior executives in pharmaceutical companies, contract research organizations and regulatory agencies) Ethics Board approval was not required. However, all study participants were provided with an information sheet about the study and were informed of all participant requirements.

All study participants provided informed verbal consent.

### Data availability

Because of the positions of study participants within their organizations and the sensitive nature of the data that they provided, confidentiality regarding that data was a specification of the study.

All study participants received assurance of the confidential nature of the study and were informed that individual responses (study data) would not be divulged to anyone apart from the research team who had entered into the confidentiality agreement with the subjects and would only be reported in an aggregated form.

## Results

### Content validity

The content validation of the 94-item developmental QoDoS^©^ (version 1) used both qualitative and quantitative methods with a 6-member expert panel of regulatory and industry professionals.

The questionnaire feedback forms (*n* = 6) from the quantitative part of the content validity demonstrated good agreement among the judges, intra-class correlation (ICC) = 0.89 (*P* < 0.0001; 95% confidence interval [CI] = 0.56, 0.99). Ninety-five percent of judges thought that the items were complete, written clearly, were relevant to decision makers and fitted well with the response options. The scale content validity index (CVI) for the 94-item developmental instrument was calculated as 0.85, confirming content validity of the QoDoS^©^. The Cronbach's alpha reliability of the ratings of six expert panel members was 0.91, indicating a high level of internal consistency. The expert panel review resulted in the reduction of the original 94 items to 76 items (version 2) and with editorial language changes being made to some of the items. The QoDoS^©^ (version 2) comprised 35 organizational- related decision-making items and 41 related to the “individual.” Content validity was successfully evaluated and the QoDoS^©^ (version 2) deemed fit for purpose and suitable for further item reduction.

### Item reduction

Item reduction was carried out using factor analysis through which relevant factors were identified.

#### Factor analysis

In this study, there were two distinct but linked stages in the factor analysis performed on the 76-item QoDoS^©^ (version 2). The first stage recruited 600 people into the study. A total of 130 took part and completed the QoDoS^©^ (version 2), of which 120 were evaluable. This sample was representative of the target population. The proprietary Survey Monkey web-based platform package was used in this study.

In addition to the factor analysis and item reduction, the research results obtained from the 120 evaluable participants provided insights into decision making from the perspective of the individual and also that of the organization in which they were/are employed. The research also allowed for comparisons to be made across the three main organizations that participated in the study; that is, regulatory agencies, pharmaceutical companies and CROs.

#### Organizational-related items

A 12-step factor analysis was performed on the 35 organizational related domains of the QoDoS^©^ (version 2) using SPSS 20. This helped to explore the underlying structure of the 35 items, to confirm appropriateness and to further develop the instrument by reducing inappropriate items that did not contribute to underlying domain factors of the instrument. Reliability testing using Cronbach's alpha and ICC were measured along with KMO and Bartlett's testing. Scree plots were generated at each factor analysis stage. The reliability statistics performed resulted in a Cronbach's alpha of 0.78 for the initial 35 Organizational items. The average ICC value for the initial organizational items was 0.77.

**Figure 4:**
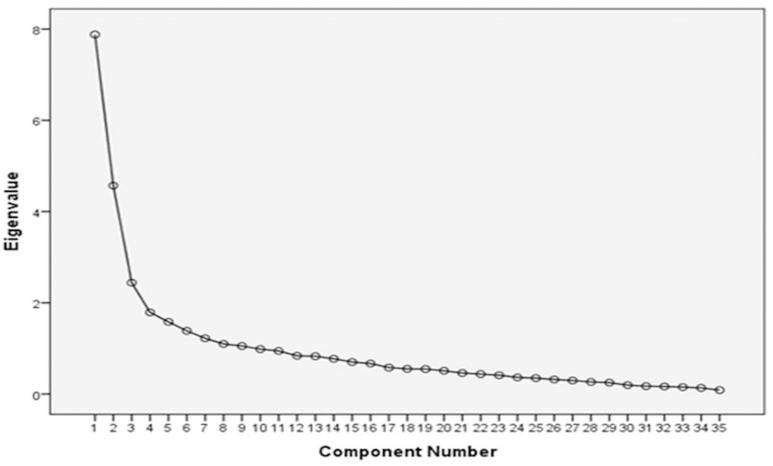
**Scree plot of 35-item organizational-related items**.

Exploratory factor analysis of the 35 organizational items was used to reduce the number of items if necessary (Figure 4). Items that failed to load on any component or had a weak loading of < 1 were removed, reducing the overall organizational-related decision-making items to a final list of 23 items. The component matrix for the extracted variables was rotated using Varimax functionality (Pallant, 2005). This Varimax rotation helped to confirm the initial structure of the scale and delivered a matrix of the factor loadings for each variable onto each of the two evident factors. Loading values with a unique value of less than 0.4 were suppressed.

#### Individual related items

A total of 11 consecutive factor analysis item reduction steps were performed on the 41 “individual related items” of QoDoS® (version 2). Reliability testing using Cronbach's alpha and ICC were measured along with KMO, scree plots and Bartlett's testing. The reliability statistics performed resulted in a Cronbach's alpha of 0.81 for the initial 41 Individual related items. The average ICC value for the initial individual items was 0.80.

Exploratory factor analysis of the 41 individual items was again used to reduce the number of items if necessary, using the same criteria as applied to the organizational items. Items that failed to load on any component or had a weak loading of < 1 were removed, reducing the overall individual related decision-making items to a final list of 22 items. The component matrix for the extracted variables was rotated using Varimax functionality. This Varimax rotation helped to confirm the initial structure of the scale and delivered a matrix of the factor loadings for each variable onto each of the two evident factors. Loading values with a unique value of less than 0.4 were suppressed.

Following the factor analysis process, the initial emergent source themes on organizational (21) and individual (22) related decision-making behavior were reviewed in order to resolve the tension between the qualitative content analysis and the outcomes of the application of statistical modeling. It was decided to retain some of the removed items which were very highly prevalent in the qualitative study. The proposed additional items were based on the research teams' personal and expert professional perspective and also with cognizance of the output from the key opinion leaders' interviews. The resultant outcome of the final factor analysis and the thorough review of the qualitative results was the delivery of the 47-item QoDoS^©^ (version 3) instrument. The instrument comprised two domains; Part 1 for organizational-level decision-making, consisting of 23 items (Figure 2) and Part 2: for individual-level approaches consisting of 24 items (Figure 3). Subsequently, each component was grouped into two separate construct domain categories. Two behavioral domain constructs were identified for the organization (*Approach* and *Culture*) and two relating to the individual (*Competence* and *Style*).

### Reliability and construct validation

Reliability and construct validity testing was performed on the QoDoS^©^ and its four component constructs using statistical methodologies complemented by additional semi-quantitative techniques (Westen and Rosenthal, 2003; Trochim, 2015). For each of the four construct categories, reliability was tested using Cronbach's alpha and ICC. For all four categories, the reliability coefficient was shown to be greater than Cronbach's alpha and ICC of 0.73. The consistency and reproducibility of the reliability findings across time as well as the different items and measurement methodologies supported the assertion that appropriate reliability was observed. The reliability, the internal consistency and the ICC of the measures observed were also indicative of the homogeneity of the component items that tapped into each of the two organizational constructs (*Approach* and *Culture*) and those for the individual (*Competence* and *Style*).

A secondary outcome of this study has been the rich insights and understanding into the decision-making practices of the 120 individuals and the organizations who participated in the factor analysis part of the study. A wealth of information was generated and this allowed several comparative reviews of perspective from that of the individual and the organization. This provided insight into the variance in distribution of results obtained from the three organizations. The initial comparative statistical analyses performed on the items of the organizational component of the QoDoS^©^ showed a high level of correlation among the regulatory authorities, pharmaceutical companies and CROs.

From the perspectives of the individual, the results showed that participants felt that they could make better decisions and that an investment in training and education would benefit them and their decision-making. There was a lack or routine usage of decision-making tools such as strengths, weaknesses, opportunities threats (SWOT) analysis and limited experience of modeling and simulation. Other perspectives that emerged were that individuals felt that they were more accountable for their decisions than the organization was at the organizational level and that professional experience was a key component that influenced decision-making. The results also showed the importance of clearly understanding the context of the decision. In total, the individual's perspective was obtained in response to 24 specific questions on their decision-making style and competence and factors that impact them.

Similarly, insights were gained from the 120 study participants on the decision-making perspective of their organization through their responses to 23 items of Part I of the QoDoS^©^. The results showed the factors influencing organizations' decision-making. These included: the culture of the organization; the lack of training in decision making provided within the organization; the internal and external competitor influences; performance of an impact analysis of decisions made; re-evaluation of a decision on the basis of new data becoming available; and transparency within the decision-making process. A variance in the distribution of the response profiles to the individual- and organizational-focused questions was evident across the three organizations involved in the research.

## Discussion

The development and initial psychometric properties of a new instrument designed to assess the quality of decision-making have been described. QoDoS^©^ is the first instrument specifically developed to assess the quality of decision making in medicines' development and review using a standardized rigorous methodology. The foundation for the QoDoS^©^ development was the earlier novel qualitative study performed which investigated the factors influencing quality decision-making (regulatory and pharmaceutical industry perspectives). (Donelan et al., 2015) The outcomes of this research identified the hallmarks of good decision-making practices that could be adopted by all stakeholders at the individual and /or organizational level, but also led to the development of the generic tool intended for use by all stakeholders. The QoDoS^©^ is a generic instrument designed to be used across all pharmaceutical research and development disciplines and regulatory agencies. An adequate number of subjects took part in the qualitative interview stage (*n* = 29) as well as in the subsequent development (120) and psychometric evaluation (*n* = 76) stages of the instrument including factor analyses, reliability and construct validation testing. The final version of the QoDoS^©^ is easy to understand straightforward and can be completed within 10 min.

At the initial development stage a robust number of emergent decision-making themes (*n* = 94) generated from interviews with industry and regulatory agency key opinion leaders were incorporated into the questionnaire development to ensure that no aspect of decision making was overlooked from the perspective of the organization or the individual. Content validation helps to examine whether a proposed measurement tool possesses the right emphasis and focus for the concept being measured and the target population. In the content validation of the 94-item instrument, both qualitative and quantitative assessment techniques were applied to ensure that the QoDoS^©^ scale had enough items and adequately covered each of the domains being measured. This is a primary validation step which helps to complement, endorse and increase the probability of obtaining high construct validation in the development of an instrument (Denzin and Lincoln, 2005). In this study, the expert panel involved in the content validation discussed and gave opinions on each questionnaire item, which led to a refined valid instrument.

We applied factor analysis, which is a well-established statistical technique for item reduction in instrument development. The strength of factor analysis lies with its ability to identify the relationships between a set of variables (items) measured or observed, in particular for those with similar (Bhatti et al., 2013) or overlapping constructs. In this study, factor analysis was used to confirm the grouping of the instrument items, which had been based on subjective opinion, through the use of mathematical modeling. It allowed the reduction of a large number of correlated variables to a more manageable number and resulting in a final quantity of 47 items in the instrument, which rendered it a more user-friendly tool. The outcome of the factor analysis was the delivery of the final version of the QoDoS^©^ instrument (version 3), capturing organizational and individual decision-making items representing the approach, culture, competence and decision-making style.

Factor analysis was followed by a demonstration of appropriate evidence of construct validity, examining convergence (evidence that different measurement methods of a construct give similar results) and discriminability (ability to differentiate the construct from other related constructs) of the QoDoS^©^ constructs. This analysis showed that the instrument possesses strong measurement properties of reliability and validity.

The secondary outcome study results have provided additional insights regarding the differing dimensions of the decision-making perspectives of organizations compared with those of individuals. For organizations, the *modus operandi*, the working environment and the shared beliefs and values of the organization appeared to be important influences. For individuals, the subjective elements of professional experience, ability, empowerment or autonomy and preference appeared to be factors influencing decision-making.

### Potential limitations

Potentially, studies of this nature have certain limitations. The participants in the qualitative phase of the research were all senior decision makers who were regarded as key opinion leaders. Whilst this cohort provided rich insights into their decision-making approaches and styles, these may not have been truly representative of personnel involved in medicines' development and review. However, if less experienced people had participated in the research, some decision-making themes that emerged in this research may not have been uncovered. Additionally, whereas the sample size achieved in the qualitative phase of the research was satisfactory, this was not the case in the quantitative phase, when only a 20% response rate was achieved despite the use of recognized techniques of follow-up including repeat emails and phone calls (Diem, 2002; Boynton, 2004; SurveyMonkey, 2015). The ideal number of participants in the final sample would have been between 350 and 760; however, this would have involved recruiting up to 3500 people, which was not an achievable target. Moreover, whilst the QoDoS^©^ research was international in nature and did include participants from several EU countries, United States, Middle East, Singapore and China, it did not include South America or Japan, and decision making in these regions may differ because of experience and culture. Finally, the lack of a validated “gold-standard” instrument could be perceived as a limitation as it precluded the opportunity for a head-to-head comparison, which in turn would have reduced the sample size requirement and also would have provided a different construct validation approach.

## Conclusions

QoDoS^©^ offers an addition to the decision-making armamentarium that provides a method to assess the quality of decision making from the perspective of the individual and the organization involved in the research, development and delivery of new medicines. It can be used to inform decision makers of factors impacting quality decision-making and be incorporated into decision-making frameworks, presenting a potential platform to add consistency, transparency and ease of communication to the subjective decision-making element. It can inform decision makers of factors impacting the methods and components of the decision making processes used by the pharma and CRO industries, as well as by regulatory authorities. As the first generic quality of decision-making measure, QoDoS^©^ has the potential to be used in a variety of health research areas and beyond.

## Author contributions

RD conceived the study, participated in its design and coordination, performed statistical analyses and helped to draft the manuscript. SW participated in the study design and coordination and helped to draft the manuscript. SS conceived the study, participated in the study design and coordination and helped to draft the manuscript.

## Funding

No funding was received by RD, SW, or SS for the design or coordination of the study, nor for the collection, analysis, and interpretation of data nor for the writing of the manuscript.

## QoDoS questionnaire

The authors do declare copyright and all reserved rights to the QoDoS questionnaire used in the research described in this manuscript.

### Conflict of interest statement

The authors declare that the research was conducted in the absence of any commercial or financial relationships that could be construed as a potential conflict of interest.

## Abbreviations

CI: confidence interval
CRO: contract research organization
EU: European Union
ICC: intra-class correlation coefficient
KMO: Kaiser-Meyer-Olkin
MTMM: multi-trait multi-method
QoDoS: Quality of Decision-Making Orientation Scheme.
